# Efficacy and sterilization resilience of silicone rubber bands as an alternative to suture ligation in feline orchiectomy

**DOI:** 10.14202/vetworld.2025.2553-2562

**Published:** 2025-08-30

**Authors:** Natnaree Raekriang, Panpicha Sattasathuchana, Naris Thengchaisri

**Affiliations:** Department of Companion Animal Clinical Science, Faculty of Veterinary Medicine, Kasetsart University, Bangkok 10900, Thailand

**Keywords:** cats, orchiectomy, silicone rubber band, sterilization, surgical efficiency, suture ligation

## Abstract

**Background and Aim:**

Suture ligation is the standard technique for feline orchiectomy, but it requires surgical expertise and is time-consuming. Silicone rubber bands (SRBs), previously used in human procedures, offer a potential alternative. This study aimed to (1) evaluate the effects of common veterinary sterilization methods on the mechanical integrity of SRBs and (2) compare the surgical outcomes of SRB versus suture ligation in feline orchiectomy.

**Materials and Methods:**

Twenty-eight: SRBs were randomly assigned to four sterilization groups: No sterilization (control), 2% chlorhexidine gluconate, autoclaving, and hydrogen peroxide gas plasma. Bands were tested for ultimate tensile strength (UTS), elongation at break, and elastic modulus using a universal testing machine. Separately, 20 healthy male cats were randomly assigned to undergo orchiectomy using either SRB ligation (n = 10) or traditional suture ligation (n = 10). Surgical time, intraoperative/post-operative complications, Glasgow composite measure pain scale scores, wound healing, and gastrointestinal (GI) symptoms were monitored for 10 days.

**Results:**

Hydrogen peroxide gas plasma sterilization preserved SRB elasticity without significantly affecting UTS, while autoclaving and chlorhexidine treatment increased stiffness and reduced elongation at break (p < 0.05). SRB ligation significantly reduced surgical time (3.06 ± 0.32 min) compared to suture ligation (4.48 ± 0.62 min; p < 0.01). There were no significant differences in post-operative pain scores, wound healing characteristics, or complication rates between groups (p > 0.05). Mild GI symptoms were observed in both groups but were not statistically different.

**Conclusion:**

SRB ligation is a viable, time-efficient, and clinically comparable alternative to traditional suture ligation in feline orchiectomy. Hydrogen peroxide gas plasma is recommended for SRB sterilization due to its minimal impact on material integrity. This technique may be especially beneficial in high-volume or resource-limited settings, offering a safe, efficient approach to feline population control. Future research should evaluate the long-term biocompatibility and broader surgical applications of SRBs in veterinary practice.

## INTRODUCTION

Orchiectomy, a standard gonadectomy procedure, is routinely performed to control cat populations and reduce the risk of hormone-induced diseases in male cats [1, 2]. Various surgical techniques have been employed to ligate the spermatic cord. Common methods include double ligation with an absorbable suture, auto-ligation, hemostatic clips, and vessel sealing devices [1]. Conventionally, suture ligation using a surgeon’s knot – a fundamental surgical technique – promotes faster wound healing and effective hemostasis [3]. However, the success of knot tying largely depends on the surgeon’s experience [4]. A previous study by Drabble *et al*. [5] found that improperly tied square knots can result in failed knots or slip knots. Failed knots may cause interoperative hemorrhage [6], which is among the most common complications during feline orchiectomy [7]. Post-orchiectomy complications such as scrotal swelling, bruising, incisional issues, abscess formation, urinary incontinence, granulomas, endocrine alopecia, eunuchoid syndrome, and behavioral changes have been reported [7, 8]. Surgical duration and suboptimal ligation techniques can prolong the inflammatory phase and delay wound healing. Given these risks, a method that ensures reliable hemostasis while reducing the operative time is warranted. Silicone rubber bands (SRBs), previously used in human female tubal ligation, may offer an effective alternative to suture ligation in feline orchiectomy by streamlining and expediting spermatic cord ligation.

Silicone rubber is valued for its inert tissue reactions and durability, including resistance to heat, chemicals, abrasion, weathering, ozone, and electrical insulating properties [9]. SRBs have been employed as an alternative ligation material in human surgical procedures [10, 11]. The application of SRBs is relatively straightforward and generally requires less time than suturing. SRBs effectively constrict blood vessels or ducts, thereby halting circulation [10]. The use of SRBs does not require extensive surgical experience because knot tying is unnecessary. In surgical practice, SRBs can be sterilized by autoclaving, ethylene oxide gas, gamma or electron-beam irradiation, or soaking in chlorhexidine gluconate. These methods may alter the mechanical properties of rubber bands, including tensile strength, elongation at break, and e-modulus [12–14].

Despite the widespread use of suture ligation in feline orchiectomy, this technique relies heavily on surgeon skill and can be time-consuming, with potential for knot failure and postoperative complications. SRBs, commonly used in human surgical applications, offer a knotless, efficient alternative for vessel ligation. While SRBs have shown promise in various medical procedures, no veterinary studies to date have evaluated their mechanical stability post-sterilization or their clinical efficacy in feline orchiectomy. Additionally, the impact of commonly used veterinary sterilization methods on the mechanical properties of SRBs, such as tensile strength, elongation, and elasticity, remains unexplored. The absence of experimental data validating SRB performance in a veterinary surgical context represents a significant knowledge gap, particularly in the search for efficient and low-skill alternatives in population control programs for companion animals.

The present study aimed to address this gap by conducting a two-part investigation. The first objective was to evaluate the effects of three commonly used sterilization methods, 2% chlorhexidine gluconate, autoclaving, and hydrogen peroxide gas plasma, on the mechanical properties of SRBs. The second objective was to compare the surgical efficiency, safety, pain response, and wound healing outcomes of SRB ligation versus traditional suture ligation in feline orchiectomy. By integrating mechanical testing with a clinical trial, this study seeks to establish the feasibility of SRB ligation as a practical, time-saving alternative to sutures in veterinary castration procedures.

## MATERIALS AND METHODS

### Ethical approval and Informed consent

The Kasetsart University Institutional Animal Care and Use Committee approved the animal study protocol under approval number ACKU68-VET-012. All experimental details and procedures were conducted and reported in accordance with Animals in Research: Reporting *in vivo* Experiments 2.0 guidelines. In addition, informed consent was obtained from all participating cat owners.

### Study period and location

The study was conducted from January 2023 to December 2024 at the Wiangsa Veterinary Clinic in Surat Thani, Thailand.

### Experimental design

**Figure 1:**
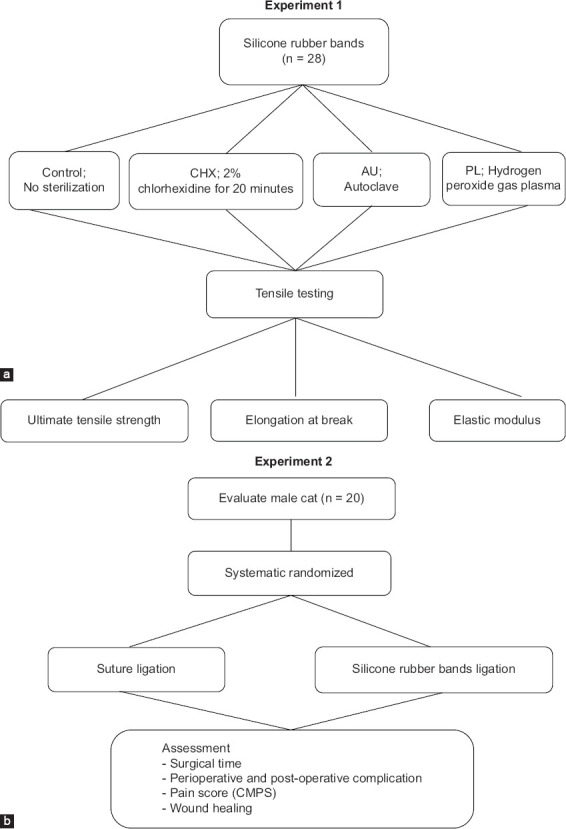
Diagram illustrating the experimental designs for the present study. (a) Experiment 1: Tensile testing of silicone rubber bands. (b) Experiment 2: Comparison of surgical outcomes between silicone rubber band and suture ligation.

The study was divided into two parts:

1. Assessment of the mechanical properties of SRBs following sterilization.
2. Clinical comparison of orchiectomy outcomes using either suture ligation or SRB ligation in male cats (Figure 1).

### Experiment 1: Evaluation of SRBs

#### Preparation and sterilization procedures

Commercial SRBs (Silicone Hemorrhoidal-Bans; Surgical Instruments; Bohemia, NY, USA) with an outer diameter of 4.8–5.0 mm, inner diameter of 1.8–2.0 mm, and length of 1.8–2.0 mm were randomly assigned to four groups (n = 7 per group):

- Control (no sterilization)
- Chlorhexidine group (CHX; 2% solution, 20 min soak) [15]
- Autoclave group (AU; 134°C, 4 min)
- Hydrogen peroxide gas plasma group (PL; 50 min, 37°C–44°C).

The 2% chlorhexidine solution was prepared by mixing 12 mL of 5% chlorhexidine gluconate (Chlorhex 5; Polipharm, Thailand) with 18 mL of distilled water (Saline Irrigate; GHP, Thailand). Autoclaving was performed in a standard hospital sterilizer (Getinge GSS67H; Sweden). Gas plasma sterilization was performed using the HO Series sterilizer (Namwiwat, Thailand). After sterilization, all bands were rinsed, air-dried, and stored under sterile conditions.

#### Mechanical testing protocol

Ring-shaped silicone specimens (2 mm thick, 5 mm long) underwent tensile testing using a universal testing machine (H50KS; Hounsfield, England) at 500 mm/min following ASTM D412 standards. The parameters measured included:

- Ultimate tensile strength (UTS)
- Elongation at break
- Elastic modulus (E-modulus).

### Experiment 2: Clinical comparison in male cats

#### Animal selection and group allocation

Twenty healthy male domestic shorthair cats (aged 8 months–3 years; body weight: 2–5 kg) were enrolled and systematically assigned to two treatment groups (n = 10 each):

- Suture ligation group
- SRB ligation group.

All cats underwent physical, hematological, and biochemical examinations (including alanine aminotransferase, aspartate aminotransferase, alkaline phosphatase, blood urea nitrogen, creatinine, and total protein). The animals were free from diseases, including feline leukemia virus, feline immunodeficiency virus, and heartworm infections, as confirmed using a commercial test kit (Snap Feline Triple; IDEXX, USA). All animals were classified as American Society of Anesthesiologists Physical Status I. Cats requiring additional medication or showing signs of illness were excluded.

#### Anesthesia and perioperative monitoring

Cats were fasted for 8 h before surgery. Premedication was administered intramuscularly (IM) using dexmedetomidine (0.01 mg/kg), buprenorphine (0.02 mg/kg), and tiletamine-zolazepam (3 mg/kg). Cefazolin (25 mg/kg IM) was given as prophylactic antibiotic. Anesthesia was maintained using 100% oxygen through face mask, and vital parameters were monitored every 5 min. Post-operative care included meloxicam (0.3 mg/kg subcutaneously [SC]) and atipamezole (0.05 mg/kg IM).

#### Surgical techniques

Closed orchiectomy was performed in all animals. A scrotal incision was made, and the testes were exteriorized without opening the parietal vaginal tunics.

**Figure 2:**
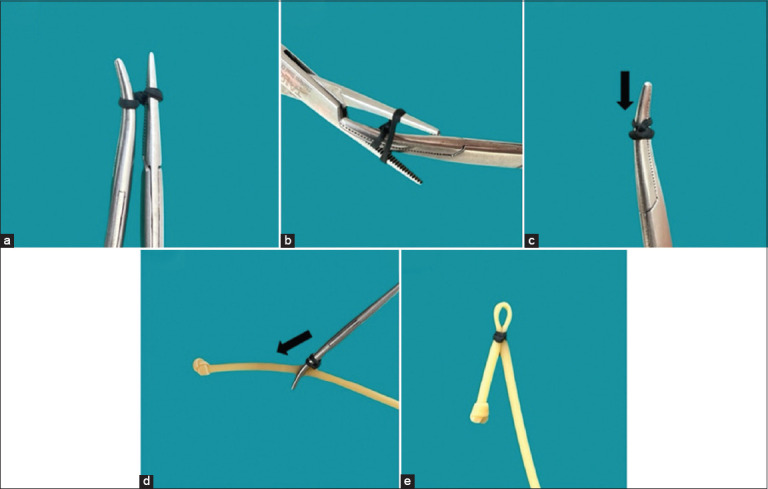
Silicone rubber band ligation technique. (a) Twist silicone rubber bands into a spiral twice with hemostats. (b) The rubber band was moved onto the curved hemostats. (c) The rubber band was pushed away from the hemostat tips by at least 0.5 cm. (d) The spermatic cord was clamped with a hemostat, and the silicone rubber bands were slid to tighten the spermatic cord. (e) The rubber band was wrapped around the spermatic cord to form a loop.

In the SRB group, hydrogen peroxide gas plasma-sterilized bands were applied using a two-hemostat twist technique (Figure 2). The rubber band was positioned using straight and curved hemostats, twisted twice, transferred, and then, slid down to ligate the spermatic cord securely. The cord was transected, and hemostasis was confirmed.

In the suture group, double ligation was performed using surgeon’s knots (three throws) with 3–0 polydioxanone sutures (Polyxanone; LongMedtech, Jiangsu, China). Scrotal incisions were left unsutured in both groups to enable wound monitoring.

#### Outcome measures and monitoring

Intraoperative data included surgical time (from incision to testis removal) and complications (e.g., hemorrhage, failed ligature). Post-operative evaluations were conducted at 6, 24, 72, 168, and 240 h and included:

- Pain scores using the Glasgow Feline Composite Measure Pain Scale (CMPS; 0–20 scale) [16].
- Wound healing, assessed through a modified Bates-Jensen wound assessment tool, as shown in Table 1 [17].
- Gastrointestinal (GI) symptoms (vomiting, diarrhea, and constipation).

**Table 1:** Criteria used for wound assessment scoring and GI complications at 6, 24, 72, 168, and 240 h postoperatively.

Item	Description of the scale
Incision	0 = Healthy, edges together
	1 = Red (approximated edges, tension, evident swelling, or induration) 2 = Red (separated edges)
Exudate type	0 = None
	1 = Bloody
	2 = Serosanguineous: thin, watery, pale red/pink
	3 = Serous: thin, watery, and clear
	4 = Purulent: thin or thick, opaque, tan/yellow, with or without odor
Exudate amount	0 = None, dry wound
	1 = Scant, moist wound but no observable exudate
	2 = Small
	3 = Moderate
	4 = Large
The skin color surrounding the wound	0 = Pink or normal for the ethnic
	1 = Bright red or blanche to touch
	2 = White or gray color or hypopigmented
	3 = Dark red, purple, or non-blanchable
	4 = Black or hyperpigmented
Scrotal edema	0 = No swelling or edema
	1 = Non-pitting edema
	2 = Pitting edema
GI complication	0 = No GI sign
	1 = One GI sign (Ex. vomiting, diarrhea, and constipation)
	2 = More than one GI sign (Ex. vomiting, diarrhea, and constipation)

GI = Gastrointestinal

Rescue analgesia (buprenorphine 0.02 mg/kg SC) was administered if CMPS >5.

### Statistical analysis

Sample size was calculated using G*Power v3.1.9.7 (http://www.gpower.hhu.de/) with an alpha of 0.05 and 80% power, based on a large effect size (d = 0.8) from prior studies on sterilization effects [18] and surgical time [19]. A minimum of six samples per sterilization group and ten animals per treatment group were required.

Data analysis was performed using GraphPad Prism v10.4.1 (https://www.graphpad.com). Normality and homogeneity were tested using Shapiro–Wilk and Levene’s tests.

- Normally distributed data were expressed as mean ± standard deviation
- Non-parametric or ordinal data were presented as median (range).

One-way analysis of variance with Tukey’s *post hoc* test was used for mechanical property comparisons. Student’s t-test compared age, body weight, and surgery time between groups. Mann–Whitney U-test was used for pain scores, wound characteristics, and GI symptoms. Statistical significance was set at p < 0.05.

## RESULTS

### Mechanical properties of SRBs after sterilization

**Figure 3:**
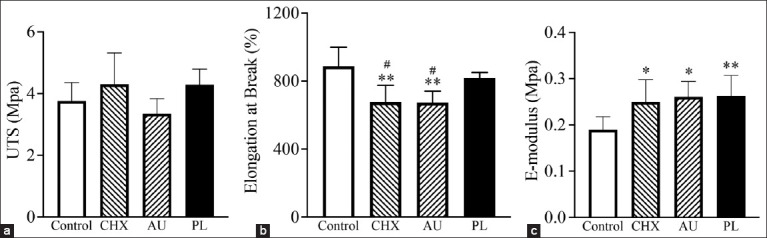
Means and standard deviations of the mechanical properties of silicone rubber bands. (a) Ultimate tensile strength, (b) elongation at break, and (c) E-Modulus in rubber bands sterilized compared with the control. Control (unsterilized), chlorhexidine, autoclave, and hydrogen peroxide gas plasma (PL) groups. * and ** indicate a significant difference between the sterilization and control groups at p < 0.05 and p < 0.01, respectively. ^#^A significant difference between the sterilization and PL groups at p < 0.05.

Figure 3 illustrates the effects of various sterilization methods on the mechanical properties of SRBs. No statistically significant differences in UTS were found between the sterilized groups and the non-sterilized control group (p = 0.05; Figure 3a). However, bands treated with 2% chlorhexidine gluconate or autoclaved showed a significant reduction in elongation at break compared with the non-sterilized control and hydrogen peroxide gas plasma groups. Notably, there was no significant difference in elongation at break between the hydrogen peroxide gas plasma group and the control group (p = 0.41; Figure 3b).

All three sterilization methods – chlorhexidine, autoclave, and gas plasma – resulted in significantly increased elastic modulus values, indicating increased band stiffness relative to the non-sterilized control (p < 0.01; Figure 3c).

### Clinical evaluation of silicone band versus suture ligation in orchiectomy

#### Comparison of age, body weight, and surgical duration

Table 2 presents the baseline characteristics and surgical time between the two ligation groups. Cats in the SRB ligation group were significantly younger (1.01 ± 0.15 years) than those in the suture ligation group (1.68 ± 0.73 years; p = 0.01). Despite this difference, all cats fell within the junior developmental stage of feline growth.

**Table 2:** Overall mean and standard deviation of age, body weight, and surgical time for cats in the SRB and suture ligation groups.

Parameter	Ligation of SRB		Suture ligation		p-value
	
	Mean	SD	Mean	SD	
Age (years)	1.01	0.15	1.68	0.73	0.01*
Body weight (kg)	3.99	0.49	4.49	0.70	0.08
Surgery time (min)	3.06	0.32	4.48	0.62	< 0.01*

* Indicates a significant difference between the SRBs ligation groups and suture ligation groups at *P <* 0.05. SD = Standard deviation, SRB = Silicone rubber band

There was no statistically significant difference in body weight between the SRB group (3.99 ± 0.49 kg) and the suture ligation group (4.49 ± 0.70 kg; p = 0.08). Surgical duration was notably shorter in the SRB group (3.06 ± 0.32 min) compared to the suture group (4.48 ± 0.62 min; p < 0.01). No intraoperative complications were reported in either group, and all animals recovered uneventfully from anesthesia.

#### Postoperative pain assessment

Pain scores were assessed using the Glasgow CMPS and are summarized in Table 3. Both groups exhibited minimal pain scores at 6 and 24 h postoperatively. By 72 h, all pain scores declined to zero and remained at zero through 240 h.

**Table 3:** The Glasgow pain score was assessed in 20 cats at 6, 24, 72, 168, and 240 h following orchiectomy using silicone rubber band ligation and suture ligation methods.

Time	Median (range)		p-value

	Ligation of silicone rubber band	Suture ligation	
6	0 (0–3)	2 (0–5)	0.19
24	0 (0–2)	0.5 (0–2)	0.35
72	0 (0–1)	0 (0–0)	1.00
168	0 (0–0)	0 (0–0)	1.00
240	0 (0–0)	0 (0–0)	1.00

There were no statistically significant differences in pain scores between the suture and SRB groups at any time point: 6 h (p = 0.19), 24 h (p = 0.34), 72 h (p = 1.00), 168 h (p = 1.00), and 240 h (p = 1.00).

#### Wound healing and postoperative complications

**Figure 4:**
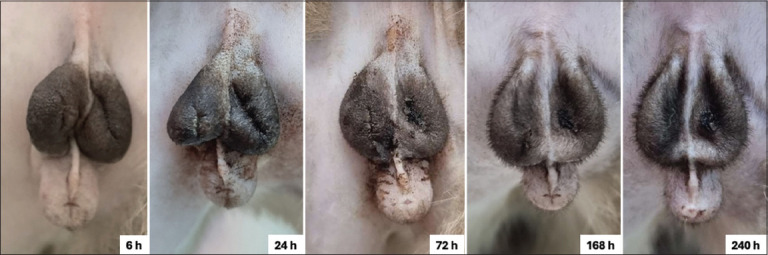
Examples of wound characteristics observed after orchiectomy with silicone rubber band ligation at 6, 24, 72, 168, and 240 h.

**Figure 5:**
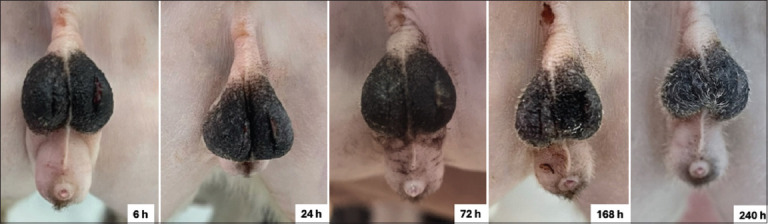
Examples of wound characteristics observed after orchiectomy with ligation of 3–0 polydioxanone absorbable sutures at 6, 24, 72, 168, and 240 h.

Wound assessment scores and GI findings are summarized in Table 4. No significant differences were observed between the two groups with respect to incision condition, exudate type and quantity, surrounding skin color, or degree of scrotal edema across all time points (6, 24, 72, 168, and 240 h; p > 0.05). Visual examination of the wound appearance further confirmed similar healing patterns in both groups (Figures 4 and 5).

**Table 4:** Summary of wound assessment score of cats in the silicone rubber band ligation and suture ligation groups during post-operative care.

Wound assessment	Group	6 h	24 h	72 h	168 h	240 h
Incision	Silicon rubber bands	2 (1–2)	1 (1–2)	1 (0–1)	0 (0–1)	0 (0–0)
	Suture	2 (1–2)	1 (1–2)	1 (1–1)	0 (0–1)	0 (0–0)
Exudate type	Silicon rubber bands	0 (0–2)	0 (0–2)	0 (0–0)	0 (0–0)	0 (0–0)
	Suture	0 (0–2)	0 (0–2)	0 (0–1)	0 (0–0)	0 (0–0)
Exudate amount	Silicon rubber bands	0 (0–1)	0 (0–1)	0 (0–0)	0 (0–0)	0 (0–0)
	Suture	0 (0–1)	0 (0–1)	0 (0–1)	0 (0–0)	0 (0–0)
Color of the skin surrounding the wound	Silicon rubber bands	0 (0–2)	0 (0–2)	0 (0–1)	0 (0–0)	0 (0–0)
	Suture	0 (0–0)	0 (0–1)	0 (0–3)	0 (0–1)	0 (0–0)
Scrotal edema	Silicon rubber bands	1 (0–1)	0.5 (0–1)	0 (0–1)	0 (0–0)	0 (0–0)
	Suture	1 (0–1)	1 (0–1)	0 (0–1)	0 (0–1)	0 (0–0)
GI complication	Silicon rubber bands	0 (0–0)	0 (0–0)	0 (0–1)	0 (0–0)	0 (0–0)
	Suture	0 (0–1)	0 (0–1)	0 (0–1)	0 (0–0)	0 (0–0)

Data are expressed as the median (range). No significant difference was found between the silicone rubber band ligation and suture ligation groups at every time point when compared using Student’s t-test. GI = Gastrointestinal

Regarding GI symptoms, two cats in the suture ligation group developed diarrhea, while one cat in the SRB group experienced vomiting. However, the incidence of GI complications was not significantly different between the groups (p > 0.05). No additional postoperative complications, such as hemorrhage, scrotal swelling, or bruising, were observed during the entire observation period in either group.

## DISCUSSION

### Mechanical integrity of SRBs post-sterilization

Suture ligation remains a widely used technique for feline castration; however, successful knot tying is essential to avoid surgical complications. This study explored the applicability of SRBs in feline orchiectomy and is the first in veterinary surgery to evaluate the effects of different sterilization methods on the mechanical properties of SRBs – highlighting a novel biomechanical perspective. Among the methods tested, hydrogen peroxide gas plasma sterilization was selected for clinical application due to its minimal impact on SRB properties. None of the sterilization techniques significantly affected the UTS of the bands, consistent with previous findings by Gupta *et al*. [20] and Gaukroger *et al*. [21], who reported no reduction in tensile strength after autoclaving vinylpolysiloxane or soaking sutures in chlorhexidine gluconate.

In contrast, both chlorhexidine and autoclave sterilization significantly reduced elongation at break, in line with findings from Daviasigamani *et al*. [22]. Although such a reduction has not been previously reported for autoclaving silicone, elevated temperatures are known to alter tensile strength, fracture properties, and elastic modulus [13]. All sterilization techniques increased the E-modulus, indicating greater rigidity and reduced elasticity. Chemical disinfectants, including aldehydes and oxidizing agents, may induce oxidative degradation of silicone materials [23]. Chlorhexidine, due to its neutral pH, is considered less damaging [14], but soaking remains a less effective method of sterilization and may still degrade silicone more than other options.

### Surgical efficiency and practical advantages

Feline orchiectomy using SRB ligation significantly reduced surgical time compared to traditional suture techniques, without increasing perioperative complications. This result aligns with human studies where silicone bands used in female sterilization led to shorter procedures than suture-based methods [24]. The SRB technique involved pre-twisting the bands using hemostats to facilitate rapid ligation, enabling secure tightening around the spermatic cord. The reduction in surgical time likely reflects the elimination of time-consuming knot tying. This observation is consistent with pony castration studies, where single ligation methods reduced procedure time compared to double ligation [25].

In some cases, additional throws are required to ensure knot security [26], adding to the duration. The SRB technique, however, allows for adjustable tightness by increasing the number of twists. Its low reliance on surgical skill makes it suitable for high-volume or resource-limited settings where time efficiency is paramount. However, excessive twisting may complicate placement, suggesting a need for technique standardization.

### Biocompatibility and postoperative outcomes

One consideration is that SRBs are non-absorbable and may persist as foreign material. In human applications, silicone is generally well-tolerated, often encapsulated by fibrotic tissue with minimal adverse reactions [27]. Polydioxanone sutures, by contrast, are absorbable and may loosen over time, with their sharp ends potentially affecting wound healing and causing complications such as hemorrhage, hematoma, or bruising. However, none of these complications were observed in this study. Scrotal edema was transient in both groups, most prominent within 24 h post-surgery, and consistent with the normal inflammatory phase of healing [28]. No other significant perioperative complications were observed.

### Pain and stress response

Postoperative pain scores did not differ significantly between groups. However, cats in the suture ligation group exhibited slightly more discomfort at 6 and 24 h. This difference may be due to the longer surgical duration and greater tissue manipulation in the suture group. Additionally, shorter anesthesia exposure in the SRB group may have contributed to reduced physiological stress and pain [29]. As a minor procedure, feline orchiectomy generally induces mild-to-moderate pain, which in this study was effectively managed with buprenorphine and meloxicam in accordance with the analgesic recommendations of the World Small Animal Veterinary Association [30] and International Society of Feline Medicine [31] guidelines.

Interestingly, outcomes in cats differed from those observed in livestock, such as calves and lambs, where external rubber ring castration is associated with prolonged inflammation and pain [32, 33]. The favorable outcomes in cats likely stem from the internal placement of the bands and appropriate analgesia, enabling faster recovery and improved animal welfare.

### Study limitations and future directions

One limitation of this study was the use of SRBs of a single size. Different dimensions may require procedural adjustments to ensure optimal tightness and placement. To the authors’ knowledge, this is the first veterinary study evaluating SRB ligation in feline neutering. Short-term results were comparable to suture ligation, supporting the biocompatibility of silicone bands. However, long-term studies are needed to assess issues such as band migration, chronic inflammation, or fibrosis when silicone is retained *in situ*.

Although SRB ligation may be less convenient than auto-ligation in some cases, it provides a safe and effective alternative. The inert tissue response and low complication rate suggest that SRBs may be valuable not only for orchiectomy but also for broader applications in ligating blood vessels or soft tissue masses in small animals.

## CONCLUSION

This study demonstrates that SRB ligation is a viable and efficient alternative to traditional suture ligation for feline orchiectomy, offering comparable surgical outcomes with significant procedural advantages. The use of hydrogen peroxide gas plasma sterilization preserved the mechanical integrity of SRBs, particularly in terms of elongation at break and elasticity, in contrast to the deterioration observed with autoclaving and chlorhexidine treatment.

Clinically, SRB ligation significantly reduced surgical time compared to suture ligation (3.06 ± 0.32 vs. 4.48 ± 0.62 min; p < 0.01), without increasing intraoperative or post-operative complications, pain scores, or impairing wound healing. Both techniques resulted in minimal discomfort and complete recovery in all cats, indicating that SRBs are both safe and effective when properly applied.

The practical applicability of SRBs is particularly relevant in high-volume or resource-limited veterinary settings where reducing operative time is essential. The technique’s simplicity, minimal dependence on surgical skill, and reduced reliance on suture material make it well-suited for widespread clinical adoption, especially in spay-neuter campaigns and welfare-based programs.

The strengths of this study include its dual experimental design (biomechanical and clinical), controlled comparison of three sterilization methods, and real-time assessment of post-operative parameters using validated pain and wound assessment tools. Importantly, this is the first veterinary study to investigate the mechanical effects of sterilization on SRBs and their surgical applicability in feline orchiectomy, adding a novel dimension to the literature.

In conclusion, SRBs, when appropriately sterilized, offer a safe, time-efficient, and cost-effective alternative to conventional suture ligation for feline orchiectomy. While short-term outcomes are promising, future studies should focus on long-term biocompatibility, potential for migration, and application across other soft tissue ligation procedures in veterinary medicine. The findings support the broader integration of SRBs into small animal surgical protocols, contributing to both surgical innovation and animal welfare enhancement.

## AUTHORS’ CONTRIBUTIONS

NT and PS: Conceptualized the study. NR: Sample collection. NT: Methodology. NR: Conducted the study. NR and NT: Formal analysis. NR: Drafted the manuscript. NT and PS: Reviewed and edited the manuscript. NR: Visualization. NT and PS: Supervised the study. All authors have read and approved the final version of the manuscript.
